# Improved yolov5 algorithm combined with depth camera and embedded system for blind indoor visual assistance

**DOI:** 10.1038/s41598-024-74416-2

**Published:** 2024-10-03

**Authors:** Kaikai Zhang, Yanyan Wang, Shengzhe Shi, Qingqing Wang, Chun Wang, Sheng Liu

**Affiliations:** 1 School of Computer Science and Technology, Huaibei Normal University, 235000 Huaibei, China; 2 Anhui Engineering Research Center for Intelligent Computing and Application on Cognitive Behavior, 235000 Huaibei, China

**Keywords:** Machine visualization, System of finding objects, YOLOv5, GhostNet, Attention mechanisms, Bidirectional feature pyramid network, Computer science, Health services

## Abstract

To assist the visually impaired in their daily lives and solve the problems associated with poor portability, high hardware costs, and environmental susceptibility of indoor object-finding aids for the visually impaired, an improved YOLOv5 algorithm was proposed. It was combined with a RealSense D435i depth camera and a voice system to realise an indoor object-finding device for the visually impaired using a Raspberry Pi 4 B device as its core. The algorithm uses GhostNet instead of the YOLOv5s backbone network to reduce the number of parameters and computation of the model, incorporates an attention mechanism (coordinate attention), and replaces the YOLOv5 neck network with a bidirectional feature pyramid network to enhance feature extraction. Compared to the YOLOv5 model, the model size was reduced by 42.4%, number of parameters was reduced by 47.9%, and recall rate increased by 1.2% with the same precision. This study applied the improved YOLOv5 algorithm to an indoor object-finding device for the visually impaired, where the searched object was input by voice, and the RealSense D435i was used to acquire RGB and depth images to realize the detection and ranging of the object, broadcast the specific distance of the target object by voice, and assist the visually impaired in finding the object.

## Introduction

According to a World Health Organization 2022 survey (*WHO world report on vision - Executive Summary*), at least 2.2 billion people worldwide have vision impairment, of whom at least 1 billion have a vision impairment that could have been prevented or has yet to be addressed. Owing to the lack of visual information support, the visually impaired face great difficulties and dangers in their daily lives; the main obstacles being object recognition and text information. Consequently, technology designed to assist them in finding daily objects indoors has attracted considerable research attention.

Although the detection and recognition of indoor objects can be challenging tasks in the field of artificial intelligence (AI)—particularly for indoor robot navigation and assisted navigation for people with visual impairment—there is a great need to develop new automated systems to fully support their daily lives indoors. The detection of specific indoor objects can be challenging as many indoor objects occupy only a few pixels of an input image, some objects may be occluded by other objects, and others may not be detected owing to lighting conditions. Enabling computers to understand their surroundings and detect and recognize objects in pictures and video streams is a valuable objective, as it is important for humans to recognize and interact with objects. However, solving the problem of recognizing objects presents a challenging task, specifically for the visually impaired.

Accordingly, target detection techniques have become a popular topic in computer vision research [1]. The techniques use a pretrained deep convolutional neural network (DCNN) to classify objects and define categories to complete the recognition, localization, and classification of objects in images or videos. Currently, mainstream target detection algorithms can be divided into two types: two-stage object detectors (e.g., region-based convolutional neural network (R-CNN) [2], Fast R-CNN [3], Faster R-CNN [4], and Mask R-CNN [5] detection methods) and one-stage object detectors (e.g., the Densebox [6], SSD series [7], and YOLO series [8–13] detection methods).

Target-acquisition algorithms require considerable processing power and memory, making their use on embedded devices difficult. The best target recognition algorithm should have an optimal balance between accuracy and speed; thus, efficient and accurate target detection algorithms that can be deployed on mobile devices are a key development trend.

Owing to the large number of redundant operations in deep learning models [14,15], several convolutional neural network (CNN) compression methods—such as network pruning [16], model quantisation [17], knowledge distillation [18], and lightweight neural networks—have been proposed, and the design of efficient neural network architectures is being developed with fewer parameters and less computational overhead. Additionally, several classical and efficient lightweight CNNs, including the mobile network [19], shuffle network [20], and GhostNet [21], have emerged.

Existing works still have limitations. Mai C et al. [22] utilized laser and ultrasonic assistance for the visually impaired; however, this method does not accurately identify specific objects. Bala M M et al. [23] employed a target detection algorithm for object detection. Barontini F et al. [24] designed a new wearable indoor navigation system that, while large, does not provide specific object recognition. Liu H et al. [25] developed a new system based on 3D point cloud instance segmentation using a solid-state LiDAR sensor for overall indoor detection and avoidance; however, it does not facilitate object recognition. In this study, a depth camera combined with the YOLO algorithm was used to simultaneously recognize specific objects and distance detection.

This study designed an indoor object detection algorithm based on a CNN to assist the visually impaired in detecting indoor objects in their daily lives. It was deployed on a Raspberry Pi device after combining it with a RealSense depth camera and voice system. The main contributions of this study are as follows.

(i) The YOLOv5 algorithm was improved by replacing the backbone network with GhostNet, adding coordinate attention, and replacing the neck network with a bidirectional feature pyramid network (BiFPN) to reduce the number of parameters and the number of computations, thus improving the accuracy of the model.
(ii) Using a RealSense D435i depth camera to obtain the precise position of an object, we combined speech recognition and speech synthesis in the indoor object detection process to obtain more accurate positional information about the object and provide information to the visually impaired.
(iii) Using a Raspberry Pi 4 B device as the core processing unit, in combination with the improved YOLOv5 algorithm, RealSense D435i depth camera, and voice input and output devices, an indoor blind object detection system was realized by performing keyword recognition. Improved convenience and mobility were also achieved.

The remainder of this paper is organized as follows. Section 2 presents the experiments and results. Section 3 presents the discussions. Section 4 presents the algorithmic deployment of embedded devices, and Sect. 5 presents the methods. Finally, the paper is concluded in Sect. 6.

## Experiments and results

### Image data acquisition

Various public datasets—such as the COCO and Google Open Image (GOI) datasets—currently contain datasets of various indoor objects; however, some of these data are easily recognizable to the visually impaired, and others are not very common. Consequently, it can be difficult for the visually impaired in indoor environments to find objects such as keys, cups, and bottles. A RealSense D435 depth camera was used for manual scanning. The data were collected for three objects: keys, cups, and bottles. The dataset obtained after manual screening, software labeling, and other steps included 1835 images and was divided into a training dataset and a test dataset in an 8:2 ratio.

### Indicators for model assessment

The algorithm classification results can be categorized into four categories: T (true) and F (false), indicating that the model prediction is true or false, respectively, or P (positive) and N (negative), indicating that the model prediction is positive or negative, respectively. Combining these predictions allows us to evaluate the performance of the model by comparing the ratios of different predictions. This evaluation method can synthesize a model’s performance across different categories and provide a quantitative assessment of its performance. The *recall*,* precision*, *mean average precision* (mAP), and model size can be used to compare the performance of each model, each of which can be expressed as follows:

1 $$P=\frac{TP}{FP+TP}$$

2 $$R=\frac{TP}{FN+TP}$$

3 $$AP={\int\:}_{0}^{1}P\left(R\right)dR$$

4 $$mAP=\frac{1}{m}\sum\:_{i=1}^{m}A{P}_{i}$$

### Comparison test

To better demonstrate the benefits of the improved model, an experiment was conducted to compare the improved YOLOv5 model with the faster R-CNN, SSD, and YOLOv5s models. The models were trained and validated using the same datasets. The comparison results of each model in terms of the mAP, model size, and number of participants are summarized in Table 1.

**Table 1:** Experimental comparison results.

Model	*P* (%)	*R* (%)	mAP (%)	Model size	Number of Parameters	Inference Speed
Faster-RCNN	59.73	88.2	83.8	110.8 MB	28,529,734	310ms
SSD	91.1	69.3	88.5	93.3 MB	26,300,756	103ms
YOLOv5s	91.7	92.6	94.3	14.4 MB	7,018,216	3.3ms
Improved YOLOv5	91.7	94.2	93.9	8.3 MB	3,775,657	4.3ms

As shown in Table 1, the YOLOv5s model in the YOLOv5 detection algorithm is a lightweight network model for the two-stage faster R-CNN and single-stage SSD, and the improved YOLOv5 model reduces the model size by 42.4%, the number of parameters by 47.9%, and the summation rate by 1.2% compared to the YOLOv5s model, while maintaining the accuracy. Comparison experiments indicate that compared with the mainstream detection algorithms, the Improved YOLOv5 model maintains a higher average accuracy and summation rate while simultaneously reducing the model size and number of parameters.

### Ablation study

Improved YOLOv5, as proposed in this paper. Firstly, in the original YOLOv5s, the improvement of the CA involves directly adding it to layer 9 of the backbone network, with data passed from the backbone network to the CA module using ordinary convolution. In contrast, in Improved YOLOv5, the integration of C3CAGhost combines GhostNet with the CA module, which is added to the backbone network, resulting in a reduction in the number of parameters added by the CA. Secondly, in the original YOLOv5s, the Neck network uses normal convolution, utilizing normal convolution data in layers 4 and 6 of the backbone network. However, in Improved YOLOv5, BiFPN is used in the Neck network to replace the original FPN + PFN, and GhostConv replaces normal convolution, utilizing GhostNet convolution in layers 4 and 6 of the backbone network. This alteration results in a reduction in the amount of data added by the BiFPN module.

An ablation experiment was conducted to verify the optimization effect of each enhancement module, and the experimental results are summarized in Table 2. It is evident that the model size is reduced by 45.1% after replacing the YOLOv5s backbone network with GhostNet; the average accuracy improves by 0.2% after adding the CA to the YOLOv5s backbone; the average accuracy improves by 0.6% after replacing the YOLOv5s neck network with a BiFPN; the size of the model decreases by 42.4% and the number of parameters decreases by 47.9% while maintaining the same accuracy after adding GhostNet to the YOLOv5s backbone network, adding the CA mechanism, and replacing the neck network with a BiFPN to obtain improved YOLOv5. The results of the ablation experiments indicate that the improved YOLOv5 model exhibits better performance, realizing the aim of reducing the number of parameters and model size while maintaining good average accuracy.

**Table 2:** Ablation experimental results.

Model	*P* (%)	*R* (%)	mAP (%)	Model size	Number of Participants
YOLOv5s	91.7	92.6	94.3	14.4 MB	7,018,216
Ghost + YOLOv5s	89.2	93.8	93.7	7.9 MB	3,683,899
CA + YOLOv5s	91.3	**95.4**	94.5	14.8 MB	7,219,992
BiFPN + YOLOv5s	90.6	93.6	**94.9**	14.8 MB	7,083,761
Improved YOLOv5	91.7	94.2	93.9	**8.3 MB**	3,775,657

In this study, the improved YOLOv5 model is based on YOLOv5s, and four additional versions (YOLOv5n, YOLOv5m, YOLOv5l, YOLOv5x) are augmented with GhostNet, CA, and BiFPN for a comparative analysis. The comparison results are presented in Table 3. The table indicates that while the model size and parameters of YOLOv5n are smaller than those of the improved YOLOv5, its P-value, R-value, and mAP-value are smaller. Conversely, YOLOv5m and YOLOv5x exhibit lower P-values than the improved YOLOv5, despite their larger model size and parameters. YOLOv5l shows a higher P-value than the improved YOLOv5; however, its model size is nearly four times larger, with parameters thirty times higher than the improved YOLOv5.

**Table 3:** Comparison results of different versions of YOLOv5.

Model	*P* (%)	*R* (%)	mAP (%)	Model size	Number of Parameters
YOLOv5n	89	92.1	92	2.4	963,701
YOLOv5m	90.3	93.1	93.7	18.1	8,722,305
YOLOv5l	92.4	92.6	94.5	32.7	115,941,185
YOLOv5x	89.2	94.7	95.4	52.2	25,598,681
Improved YOLOv5	91.7	94.2	93.9	8.3	3,775,657

## Algorithmic deployment of embedded devices

### Generalization and the normalization done on the model to be deployed

Generalization is the ability of a model to perform well on new and previously unseen data. Techniques for generalization aim to mitigate overfitting to the training data, enhancing the model’s adaptability to diverse data distributions and scenarios, thereby improving its performance on new data. Normalization aims to standardize the range of input data values, facilitating more effective model training and performance improvement. This process mitigates scale inconsistency, gradient vanishing, and gradient explosion issues, thereby enhancing the performance and stability of deep learning models.

In YOLOv5, data augmentation, feature pyramid networks, and several regularization techniques including weight decay and Dropout are employed to mitigate model overfitting and enhance generalization. The batch normalization (BN) technique is utilized in YOLOv5 to mitigate Internal Covariate Shift and expedite the convergence of model training.

This study introduces the GhostNet module, Coordinate Attention, and BiFPN module in the improved YOLOv5 framework. GhostNet enhances the model’s generalization ability by introducing the Ghost Module, lightweight design, data augmentation, and regularization strategy. Attention enhances the model’s generalization ability by modeling spatial relationships, enhancing flexibility, facilitating feature fusion, and implementing data augmentation. This enables the model to better adapt to diverse data distributions and application scenarios, thereby improving its performance and stability. The BiFPN module enhances the model’s generalization ability through multi-scale feature fusion and bi-directional information transfer. The GhostNet and BiFPN modules employ BN to normalize the improved YOLOv5 model.

Despite the differences in system architecture, instruction set, processing speed, storage capacity, and computational power between embedded systems and the platforms used for model training, deep learning models are deployed in embedded systems.[26] However, in this study, the same deep learning architecture enables the trained model to be directly deployed on the embedded device. This deployment does not compromise the generalization and normalization of the model. Models deployed on embedded systems only perform target predictions and do not require extensive computational resources.

### Embedded components embedded composition and visualization results

The improved YOLOv5 model, combined with a depth camera and a Raspberry Pi 4 B device, was used to design a portable indoor object-finding system. The system uses a RealSense D435i depth camera to acquire RGB and depth images. The Raspberry Pi 4 B device is used as the core processing unit for processing the image data to run the improved YOLOv5 model, speech recognition, and synthesis output; moreover, the small power amplifier board includes a microphone, speaker, and amplifier components to realise speech reception and language output. The specific components are listed in Table 4.

**Table 4:** Functions of the main components of the blind tracing system.

Device Name	Functionality	
Embedded System (Raspberry Pi 4B-8G RAM)	Process image data, run Improved YOLOv5 algorithm and language recognition, synthesis, and output	
Depth Camera (Intel RealSense D435i)	Acquire RGB image and depth image	
Compact amplifier module (microphone, speaker, amplifier)	Connect to Raspberry Pi 3.5 mm connector to provide voice reception, output voice amplification, and voice output	
Raspberry Pi UPS (18650*2,2600mAh)	Provide power to the Raspberry Pi, which reads the remaining power via the IIC	

**Fig. 1:**
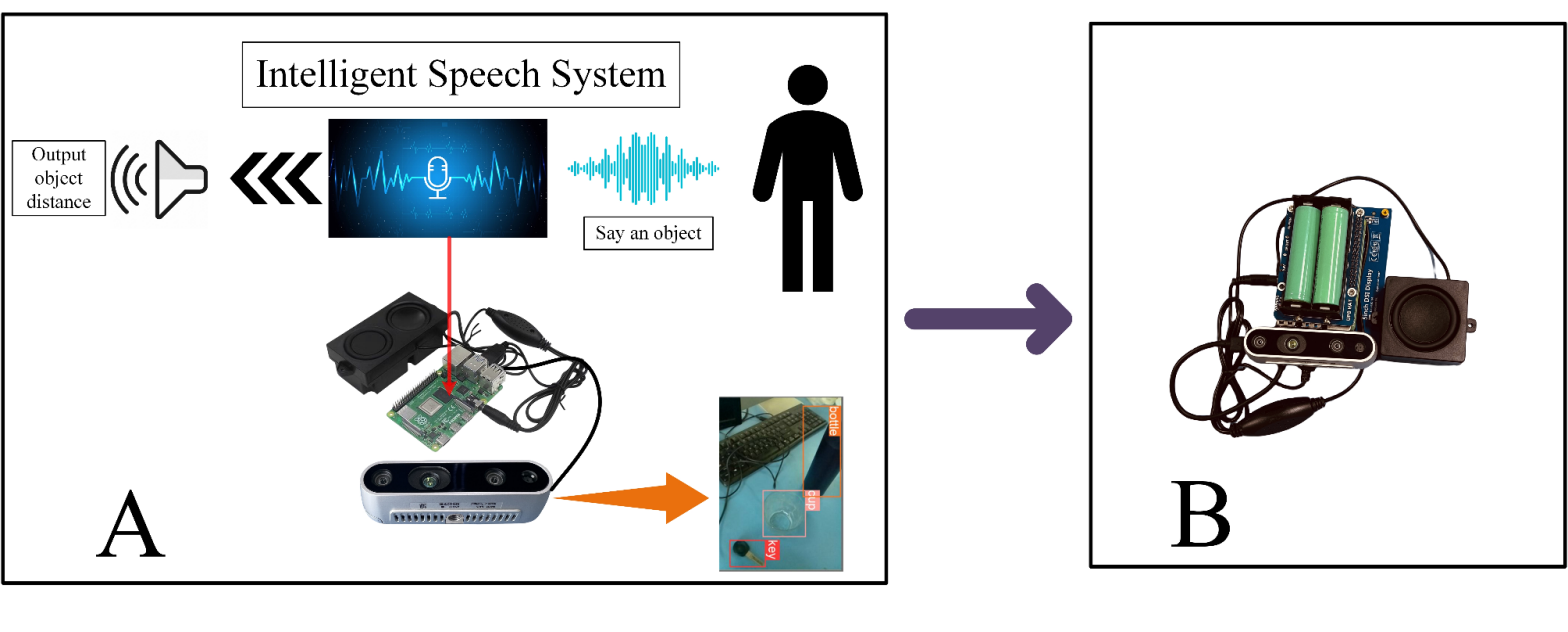
Schematic diagram of the principle of the object-finder system for the visually impaired (A. working principle, B. actual assembly).

RGB information is acquired using the Realsense D435i camera from the area in front of visually impaired individuals. This data is used as input for the Improved YOLOv5 model deployed on the Raspberry Pi, facilitating object detection and position calculation. Upon a successful match between the detected target and the speech text information, the Raspberry Pi will output the depth distance(using the depth information obtained by the RealSense D435i depth camera) of the object via a small amplifier module. If the match is unsuccessful, the compact amplifier module will broadcast that no objects are being searched for in this area.As in Fig. 1.

**Fig. 2:**
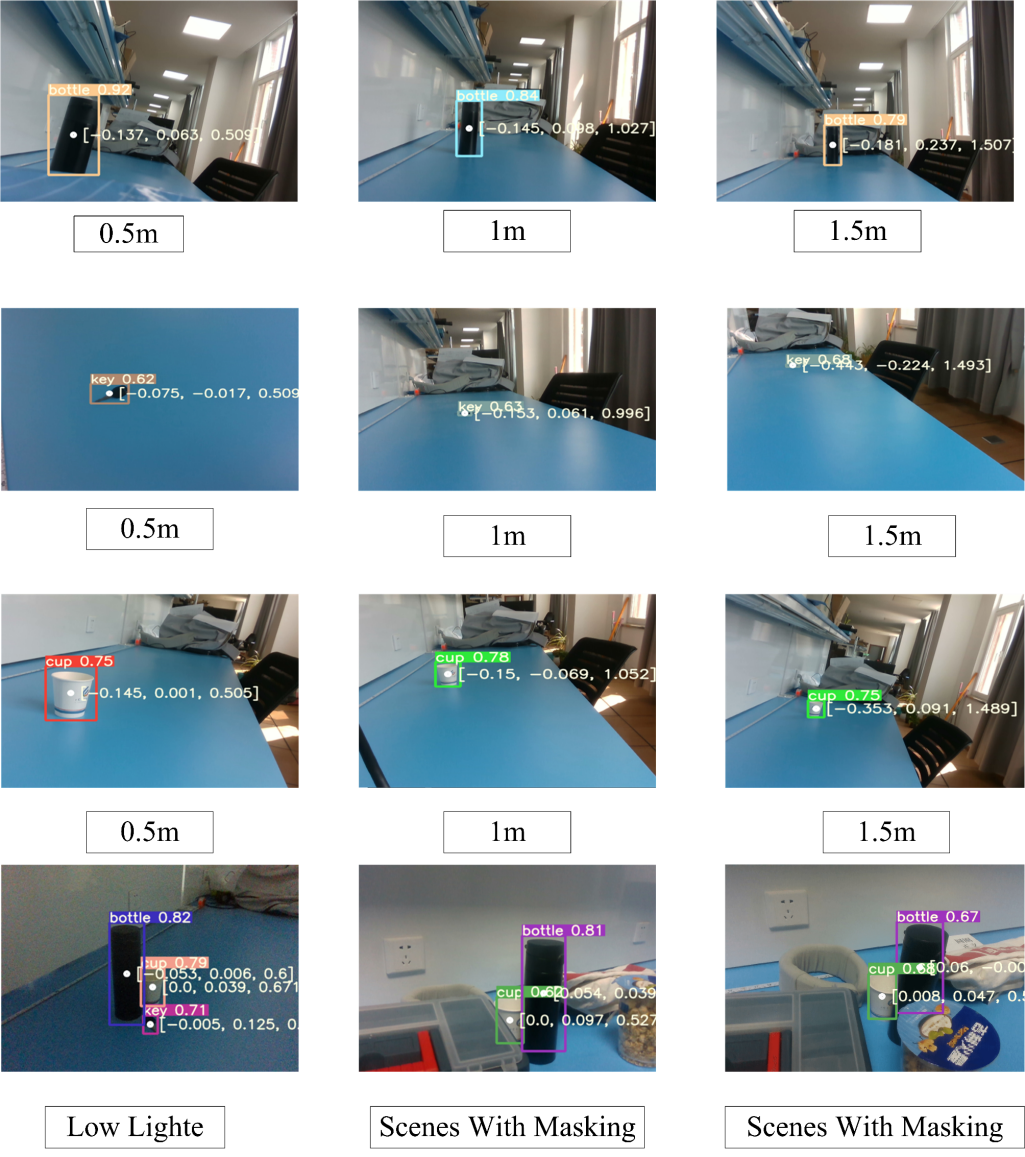
Visualization of actual measurements.

Utilizing the RealSense D435i camera, object recognition and distance detection were conducted. The depth of the object recognized by the depth camera was compared with the depth obtained from experimental measurements to determine the accuracy of the depth estimation. Figure 2 illustrates the experimental setup, where three types of objects were used to conduct object recognition and ranging experiments in the range of 0.5–1.5 m. Additionally, object detection experiments were performed in complex scenes with masking and low lighting conditions. Here, the output of the depth camera provides object position information in the form of $[x,y,z]$ coordinates, where $x$ and $y$ represent the plane coordinates and $z$ represents the depth coordinates.

## Discussion

### Key benefits

The primary feature of this project is to improve the YOLOv5 algorithm and port it to an embedded device to create an indoor object-finding system designed to assist the visually impaired in conjunction with RealSense D435i and a voice system.

The YOLOv5 algorithm was improved by replacing the backbone network to reduce the number of parameter operations, adding an attention mechanism, and modifying the neck network to ensure the accuracy of target detection while reducing the number of parameter operations. This enhanced algorithm is successfully ported to an embedded system, which completes the design of a portable indoor assistive instrument for the visually impaired based on the YOLOv5 algorithm. The improved YOLO method demonstrates a better ability to detect objects indoors, and experiments indicate a 42.4% reduction in model size, a 47.9% reduction in the number of parameters, and a 1.2% increase in recall rate.

The RealSense D435i is used in embedded devices to accurately acquire both color images and depth information of objects within a range of 0.2–10 m. It only needs to be connected to the embedded device via USB and does not need to consume the computing memory of the main processor of the embedded device, making it suitable for indoor scenarios involving the visually impaired. In addition, the RealSense D435i is small, portable, and compatible with various operating systems and development platforms.

Embedded systems offer the advantage of audio development and support various speech recognition engines, which can easily realize real-time speech input, language synthesis, and language output, providing multiple language support. In this study, an online speech engine is used in the Raspberry Pi system to achieve keyword recognition with high accuracy, and a microphone and amplifier module are connected through the 3.5 mm audio interface of the Raspberry Pi to realize speech reception and speech output.

### Practical issues

The indoor finding aid for the visually impaired has the advantages of accurate detection and portability. However, this study has shortcomings and limitations in practice. The visually impaired may have the following problems during practical application.

Dataset issues: The dataset created for this study is limited to the objects collected during indoor; items not included in the dataset cannot be recognized. Further data collection for each type of object is required to expand the dataset to cover additional object types.

Lighting issues: The visually impaired cannot perceive the brightness of light autonomously, and in their actual operations, there may be insufficient or no light to detect objects. The system can be combined with a brightness sensor to sense room brightness and verbal prompts to ensure adequate light illumination in the environment.

Accent issues: In different regions with different accents and dialects, keyword detection may not be sufficiently accurate to find the target object. According to the characteristics of different pronunciations, the corresponding voice recognition module can be added to solve the problem of accent recognition.

### Next steps

(i) Using infrared sensors to monitor the indoor movements of the visually impaired, generate action track maps, accurately locate the position of blind individuals indoors, and, simultaneously increase the system’s memory function of specific locations of objects can quickly and accurately provide specific locations of objects, assisting them to move freely indoors and retrieve objects.
(ii) The use of speech recognition models in deep learning to improve the accuracy of language recognition in complex language environments and noisy external environments and to improve the understanding of complex utterances can better assist the visually impaired in their daily lives. Furthermore, the system can be combined with embedded devices to realize more functions.
(iii) Adapting the system to more complex outdoor environments can help the visually impaired recognize and understand their environment. This may involve detecting slippery surfaces or dangerous roads and providing appropriate warnings and reminders to assist them to walk.

## Methods

**Fig. 3:**
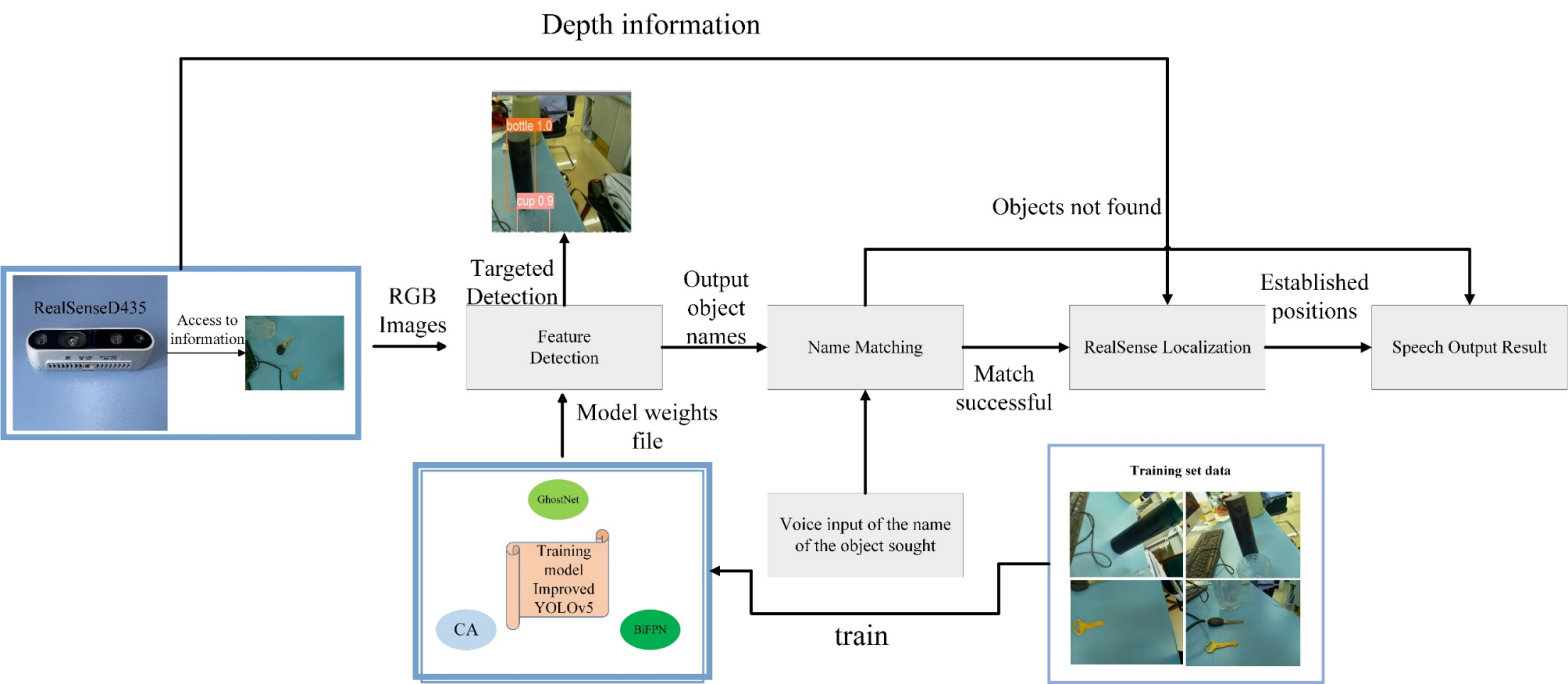
Improved YOLO indoor object-finding system for the visually impaired.

A RealSense D435i depth camera developed by Intel Corporation was used to acquire images of the indoor objects. The proposed indoor object-finder system for the blind is shown in Fig. 3. If the name of a detected object matches a keyword from the speech input, the distance is measured using the RealSense D435i, and the name of the object and the distance are output by the speech module.

### Improved yolov5 network architecture

The YOLOv5 algorithm belongs to the you only look once (YOLO) series of algorithms released in June 2020 by Ultralytics, an AI research organization in the U.S. The YOLOv5 algorithm transforms the target detection task into a regression problem and uses the CNN to process the input image end-to-end to rapidly detect the target in the image. It is based on the PyTorch framework, which has fewer model parameters and is easier to deploy on mobile terminals and embedded devices.

The YOLOv5-v6 release was used in this study. Depending on the number of feature extraction modules and convolutional kernels in the backbone, there are five versions—YOLOv5s, YOLOv5m, YOLOv5m, YOLOv5l, and YOLOv5x—whose model sizes and parameters gradually increase. In this study, a lightweight object detection model was investigated for application in real-world environments to strike a balance among accuracy, model size, and parameter size. The YOLOv5s model was chosen as the base model for the subsequent study. The basic framework structure of this version of YOLOv5 comprises four main quadratic layers—that is, the input, backbone, neck, and prediction layers.

**Fig. 4:**
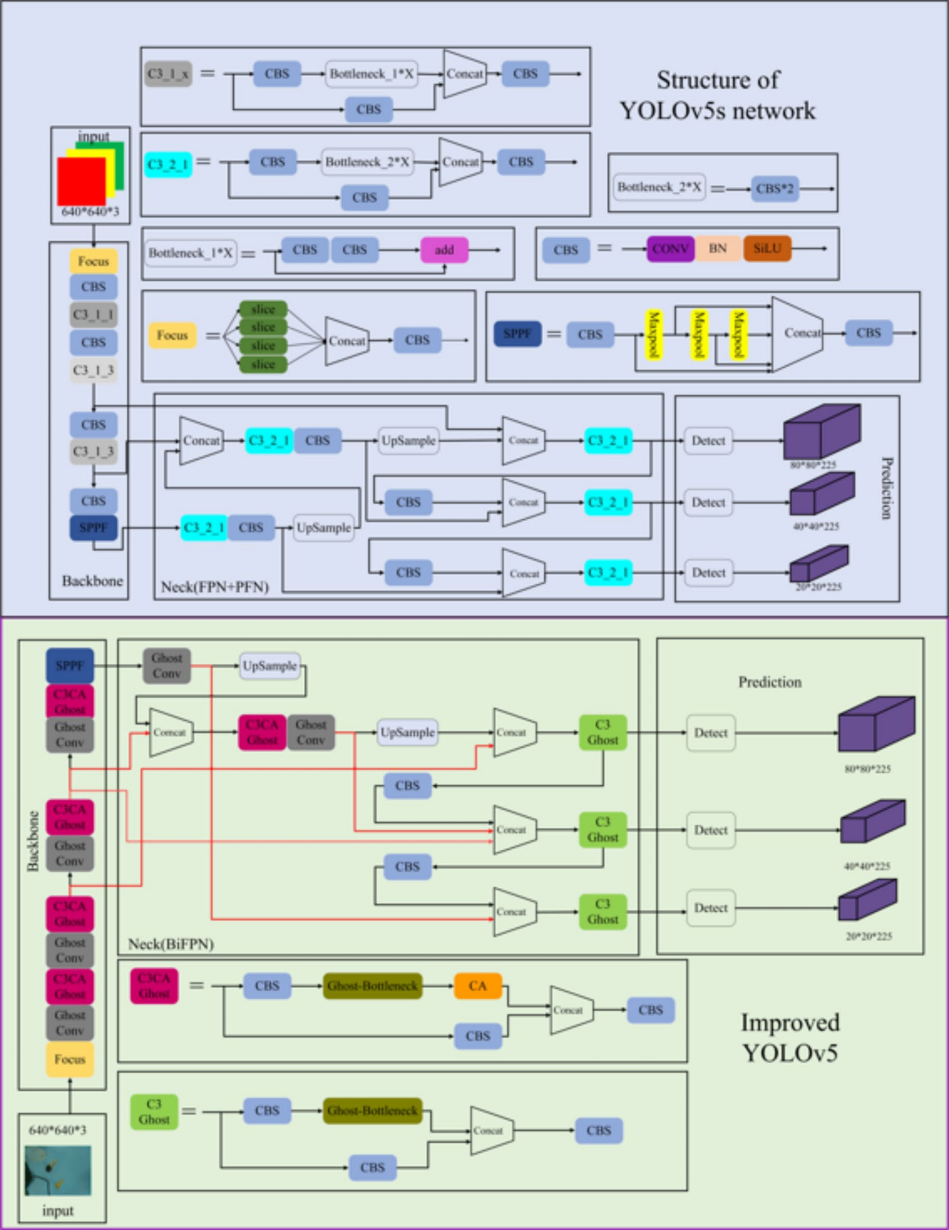
YOLOv5 structure.

In indoor environments, morphological differences between objects and severe mutual overlapping and occlusion can increase the difficulty of detection, with object detection accuracy being low. To address this problem, an object detection method based on the attention mechanism, BiFPN [28], and improved YOLOv5 model was proposed as follows:

(i) To address the problem of large parameters in the YOLOv5-based object detection model, the GhostNet module was proposed, a method that could considerably reduce the number of model parameters and model complexity.
(ii) To improve the detection accuracy of the lightweight YOLOv5 model, this study proposed the integration of a lightweight coordinate attention (CA) [27] mechanism into the backbone feature extraction network to obtain more feature information and equip it with a more powerful feature extraction capability. The CA mechanism was integrated into the Ghost Bottleneck structure of the backbone to obtain a lightweight feature extraction network.
(iii) The BiFPN [28] was used to replace the original neck layer (Neck) to further improve the YOLOv5 model accuracy. The proposed lightweight model could be applied to mobile devices with low computational power to achieve rapid and accurate target recognition in indoor environments. The green portion of Fig. 4 shows the network structure diagram of the improved YOLOv5 model.

Notably, the recently released YOLOv9 [29] model demonstrates some improvement in average detection accuracy and speed compared to YOLOv5. It comes with a significant increase in network model parameters. For instance, the YOLOv5 model comprises approximately 7.03 million parameters, whereas the YOLOv9 consists of approximately 51 million parameters. Moreover, the YOLOv5 algorithm has demonstrated effectiveness and high performance in various indoor object recognition tasks, encompassing categories such as fruits [30], chairs [31], and waste [32]. Hence, to enable deployment of the model on devices lacking GPU support, the mature and versatile YOLOv5 is selected as the foundational framework in this study.

#### Ghostnet

**Fig. 5:**
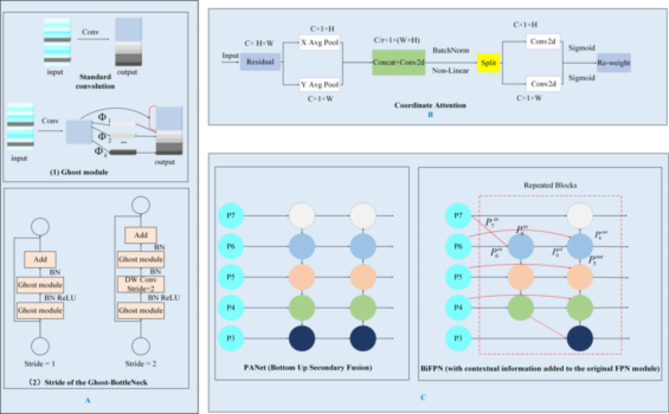
(A) GhostNet network architecture, (B) CA structure, (C) PANet and BiFPN network structure.

GhostNet is a lightweight feature extraction network proposed in 2020 [21]. The network aims to improve model accuracy and operational efficiency and help solve the challenge of using CNNs in embedded devices. The image features are processed by the DCNN, where redundant features are called ghosts. Based on the similarity between the feature map channels of the network, a linear transformation can be introduced between the channels instead of the original convolutional operation to reduce the network parameters and computational overhead while maintaining the original number of feature map channels. Part (1) of A in Fig. 5 shows the standard convolution and ghost module convolution processes. With similarly-sized input and output feature maps, the computational overhead of the ghost module is significantly lower than that of normal convolution, which yields more feature information with less computational overhead without negatively affecting the performance.

The Ghost Bottleneck can be constructed by stacking two ghost modules, as shown in (2) of A (Fig. 5). When the step size is 1, the first ghost module acts as an expansion layer, increasing the number of channels and the curvature dimension. The second ghost module is used to reduce the number of channels and feature dimensions and is matched to a shortcut path, which connects the inputs and outputs of the two ghost modules. With reference to the MobileNetV2 structure, we introduced the ReLU [33] activation function in the second ghost module as well as the BN [34] and ReLU activation functions after each layer. When the step size is 2, the shortcut path comprises a downsampling layer and a deep convolution with a step size of 2, with the deep separable convolution helping to reduce the number of channels.

#### Coordinate attention

Attentional mechanisms first emerged in neuroscience to explain how the human brain selects and focuses attention on processing information. Studies have demonstrated that with complex tasks, people tend to focus on a subset of relevant information, giving it more attention and processing power while ignoring other irrelevant information. This mechanism allows people to process information efficiently and extract the important features. Attentional mechanisms have been shown to be useful in various computer vision tasks.

Iterations of the YOLOv5 algorithm accumulate a large volume of redundant background information, with the feature information of common objects being easily lost, resulting in poor detection accuracy. To solve this problem, we introduced a lightweight CA and integrated it into the YOLOv5 model of the backbone feature extraction network. The CA module aims to enhance the expressive ability of a mobile network to learn features. It can take any intermediate feature tensor $X=\left[{x}_{1},{x}_{2},\dots\:,{x}_{c}\right]\in\:{R}^{C\times\:W\times\:H}$as an input, and obtain an output feature tensor $Y=\left[{y}_{1},{y}_{2},\dots\:,{y}_{c}\right]\in\:{R}^{C\times\:W\times\:H}$with the same size as the input, where $C$ denotes the number of channels; $H$ and $W$ denote the height and width of the input feature map, respectively. As shown in Fig. 5, the CA module is divided into two steps—that is, coordinate information embedding and coordinate attention generation—which encode the channel relationship and long-range dependency, respectively, using precise positional information.

When the size of the input feature map is $C\times\:H\times\:W$, to focus on the height and width of the image and encode the exact positional information, the input features can be divided into two directions: the height and width for global average pooling using pooling kernels of dimensions $\left(H,1\right)$ and $\left(1,W\right)$, respectively. To obtain the ${Z}^{h}$and ${Z}^{w}$ features in both the height and width directions and distance dependence in one spatial direction and exact positional information in the other spatial direction, as expressed in Eqs. (1) and (2), the feature maps obtained in the height and width directions of the global perceptual field can be stitched together, and the F1 transform can be performed in a $1\times\:1$ shared convolutional kernel to generate the intermediate feature map *f*, as shown in Eq. (3). The feature map *f* can then be convolved using a $1\times\:1$ convolution kernel based on the original height and width, with the attention weights in the height and width directions being obtained based on the activation function, as shown in Eqs. (4) and (5), respectively. Finally, the attention weights in the height and width directions can be multiplied and weighted using the original feature maps to obtain the attention weight feature maps in the width and height directions, as expressed in Eq. (6).

5 $${\:Z}_{c}^{h}\left(h\right)=\frac{1}{W}\sum\:_{0\le\:i\le\:W}{x}_{c}\left(h,i\right)$$

6 $${Z}_{c}^{w}\left(w\right)=\frac{1}{H}\sum\:_{0\le\:j\le\:W}{x}_{c}\left(j,w\right)$$

7 $$f=\delta\:\left({F}_{1}\left(\left[{Z}^{h},{Z}^{w}\right]\right)\right)$$

8 $${g}^{h}=\sigma\:\left({F}_{h}\left({f}^{h}\right)\right)$$

9 $${g}^{w}=\sigma\:\left({F}_{w}\left({f}^{w}\right)\right)$$

10 $${y}_{c}\left(i,j\right)={x}_{c}\left(i,j\right)\times\:{g}_{c}^{h}\left(i\right)\times\:{g}_{c}^{w}\left(j\right)$$

#### Bidirectional feature pyramid network

Feature pyramids have emerged as feature-fusion frameworks for detecting objects at different scales. However, traditional feature pyramid structures—such as those in a feature pyramid network (FPN) [35] or PANet architecture [36]—add different input features during fusion. Because these input features have different resolutions, their contributions to the fused features are different. To address this, we used a BiFPN to replace the original FPN + PANet structure in the YOLOv5 neck.

The BiFPN is a bidirectional weighted feature pyramid network that enables easy and fast fusion of multiscale features. It achieves cross-scale connectivity by constructing bidirectional channels through weighted and bidirectional connections—that is, top-down and bottom-up structures—and directly fuses features in the feature extraction network with relative-sized features in the bottom-up paths. The weighted fusion approach uses a fast normalized fusion technique, which accelerates the training expression of the YOLO network. The improved network structure is shown in Fig. 5, with the fast normalized fusion in the BiFPN structure being expressed as follows:

11 $$O=\sum\:_{i}\frac{{\omega\:}_{i}*{I}_{i}}{\epsilon+{\sum\:}_{j}{\omega\:}_{j}}$$

12 $${P}_{6}^{td}=Conv\left(\frac{{\omega\:}_{1}*{P}_{6}^{in}+{\omega\:}_{2}*Resize\left({P}_{7}^{in}\right)}{\epsilon+{\omega\:}_{1}+{\omega\:}_{2}}\right)$$

13 $${P}_{6}^{out}=Conv\left(\frac{{\omega\:}_{1}^{{\prime\:}}*{P}_{6}^{in}+{\omega\:}_{2}^{{\prime\:}}*{P}_{6}^{td}+{\omega\:}_{3}^{{\prime\:}}*Resize\left({P}_{5}^{out}\right)}{\epsilon+{\omega\:}_{1}^{{\prime\:}}+{\omega\:}_{2}^{{\prime\:}}+{\omega\:}_{3}^{{\prime\:}}}\right)$$

where $Resize$ denotes an up-sampling or down-sampling operation and $\omega\:$ denotes a weight parameter that determines the importance of additional features.

### Object distance and position acquisition

This study used an Intel RealSense D435i depth camera with a depth map resolution of 1280 × 720 pixels, a color resolution of 1920 × 1080 pixels, and a depth detection range of 0.2–10 m. The Intel RealSense D435i offers the advantages of rapidly processing data, adapting to light changes, and capturing accurate depth information. It is small, does not require an external power supply, and can be connected to the Raspberry Pi device via its own USB interface, which is suitable for data transfer on mobile devices. The built-in parameters of the Intel RealSense D435i are listed in Table 5.

**Table 5:** Parameters in RealSense D435i camera.

Camera	Parameters
$R$	$$\left(\begin{array}{cc}0.999942&\:\begin{array}{cc}0.00685799&\:-0.00835137\end{array}\\\:\begin{array}{c}-0.00684949\\\:0.00835834\end{array}&\:\begin{array}{cc}\begin{array}{c}0.999976\\\:-0.000988772\end{array}&\:\begin{array}{c}0.00104604\\\:0.999965\end{array}\end{array}\end{array}\right)$$
$T$	$$\left[\begin{array}{c}0.0148185\\\:0.000240078\\\:0.000289946\end{array}\right]$$
${G}_{RGB}$	$$\left[\begin{array}{cc}615.842&\:\begin{array}{cc}0&\:323.826\end{array}\\\:\begin{array}{c}0\\\:0\end{array}&\:\begin{array}{c}\begin{array}{cc}615.943&\:242.337\end{array}\\\:\begin{array}{cc}0&\:1\end{array}\end{array}\end{array}\right]$$
${G}_{DEPTH}$	$$\left[\begin{array}{cc}385.7&\:\begin{array}{cc}0&\:320.823\end{array}\\\:\begin{array}{c}0\\\:0\end{array}&\:\begin{array}{c}\begin{array}{cc}385.7&\:239.159\end{array}\\\:\begin{array}{cc}0&\:1\end{array}\end{array}\end{array}\right]$$

Where $R$ and $T$ denote the external parameters of the RealSense D435i, which are used to describe the position and orientation of the camera in the world coordinate system. ${G}_{RGB}$ and ${G}_{DEPTH}$ denote the external parameters of the RealSense D435i, which are used to perform camera calibration, geometric corrections, and compute depth information.

#### Depth camera calibration

In addition to camera calibration boards included with the hardware, Intel provides the official Intel Realistic Dynamic Calibration software and camera calibration boards, for camera calibration. These methods and software are used to calibrate the camera. Upon completion of calibration, the resulting calibration parameters are applied to replace the pre-calibration parameters, enhancing accuracy.

#### Object localization

**Fig. 6:**
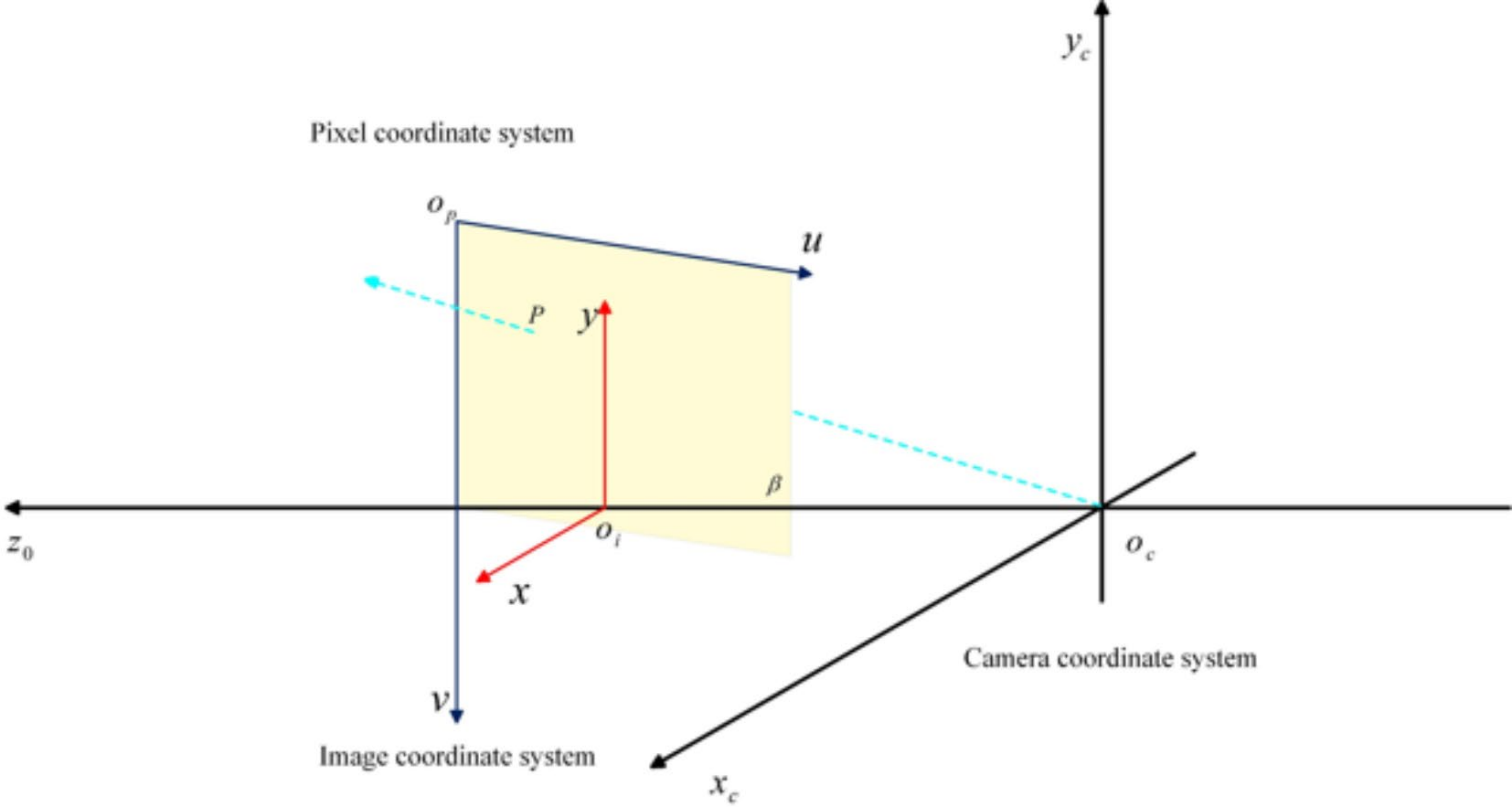
Camera coordinate transformation model. $\left\{{x}_{c},{y}_{c},{z}_{c}\right\}$ denotes the camera coordinate system with origin ${O}_{C}$; $\left\{x,y\right\}$ denotes the image coordinate system with origin ${O}_{i}$; $\{u,v\}$ denotes the pixel coordinate system with origin ${O}_{p}$; point *P* denotes the intersection of the projected light ray, ${O}_{C}\:{P}_{C}$, being the plane of the image coordinate system; $\beta\:$ denotes the focus length, ($mm$).

As shown in Fig. 6, we aim to determine the exact position of an object in the world coordinate system. To achieve this goal, we first need to compute the transformation relationship of the centre of the object with respect to the camera coordinate system $\left\{{x}_{c},{y}_{c},{z}_{c}\right\}$, and then determine the transformation relationship from the camera coordinate system to the image coordinate system $\left\{x,y\right\}$ and from the image coordinate system to the pixel coordinate system $\left\{u,v\right\}$. Here, the coordinate system $\left\{x,y\right\}$ is parallel to the plane consisting of ${x}_{c}$ and ${y}_{c}$ in the coordinate system $\left\{{x}_{c},{y}_{c},{z}_{c}\right\}$, whereas the origin of the coordinate system $\left\{u,v\right\}$ is located in the upper-left corner of the image. By applying the rigid body transformation matrix, we can transform the localization point to point ${P}_{c}$ under the camera coordinate system, and based on the similar triangle principle of projected rays, we can derive the transformation relationship between the coordinate system $\left\{{x}_{c},{y}_{c},{z}_{c}\right\}$ and the coordinate system $\left\{x,y\right\}$, expressed as follows:

14 $${\:z}_{c}=\left[\begin{array}{c}x\\\:y\\\:1\end{array}\right]=\left[\begin{array}{c}\beta\:\\\:0\\\:0\end{array}\:\:\:\:\begin{array}{ccc}0&\:0&\:0\\\:\beta\:&\:0&\:0\\\:0&\:1&\:0\end{array}\right]\left[\begin{array}{cc}R&\:t\\\:0&\:1\end{array}\right]\left[\begin{array}{c}\begin{array}{c}{x}_{c}\\\:{y}_{c}\end{array}\\\:{z}_{c}\\\:1\end{array}\right]$$

Because the image coordinate system has a translation relationship with the pixel coordinate system, the two have another transformation relationship as follows:

15 $$\left[\begin{array}{c}u\\\:v\\\:1\end{array}\right]=\left[\begin{array}{ccc}{\beta\:}_{x}&\:0&\:{u}_{0}\\\:0&\:{\beta\:}_{y}&\:{v}_{0}\\\:0&\:0&\:1\end{array}\right]$$

where ${\beta\:}_{x},{\beta\:}_{y},{u}_{0},{v}_{0}$ denote the in-camera parameters from Table 5; ${\beta\:}_{x}=\frac{1}{{d}_{x}},\:{\beta\:}_{y}=\frac{1}{{d}_{y}}$ denote the normalized focal lengths in pixels on the *x*- and *y*-axes of the image coordinate system; ${d}_{x},{d}_{y}$ denote the dimensional sizes in pixels per unit on the *u-* and *v*-axes of the pixel coordinate system, respectively. Associative formulations of Eqs. (10) and (11) yield the following:

16 $${z}_{c}\left[\begin{array}{c}u\\\:v\\\:1\end{array}\right]=\left[\begin{array}{c}{\beta\:}_{x}\\\:0\\\:0\end{array}\begin{array}{c}0\\\:{\beta\:}_{y}\\\:0\end{array}\begin{array}{c}{u}_{0}\\\:{v}_{0}\\\:1\end{array}\right]\left[\begin{array}{c}{x}_{c}\\\:{y}_{c}\\\:{z}_{c}\end{array}\right]$$

That is, the conversion formula for the camera and pixel coordinate system, where ${x}_{c},{y}_{c}$ can be expressed as follows:

17 $$\left\{\begin{array}{c}{x}_{c}=\frac{{z}_{c}\cdot\:\left(u-{u}_{0}\right)}{{\beta\:}_{x}}\\\:{y}_{c}=\frac{{z}_{c}\cdot \:\left(v-{v}_{0}\right)}{{\beta\:}_{y}}\end{array}\:\right.$$

**Fig. 7:**
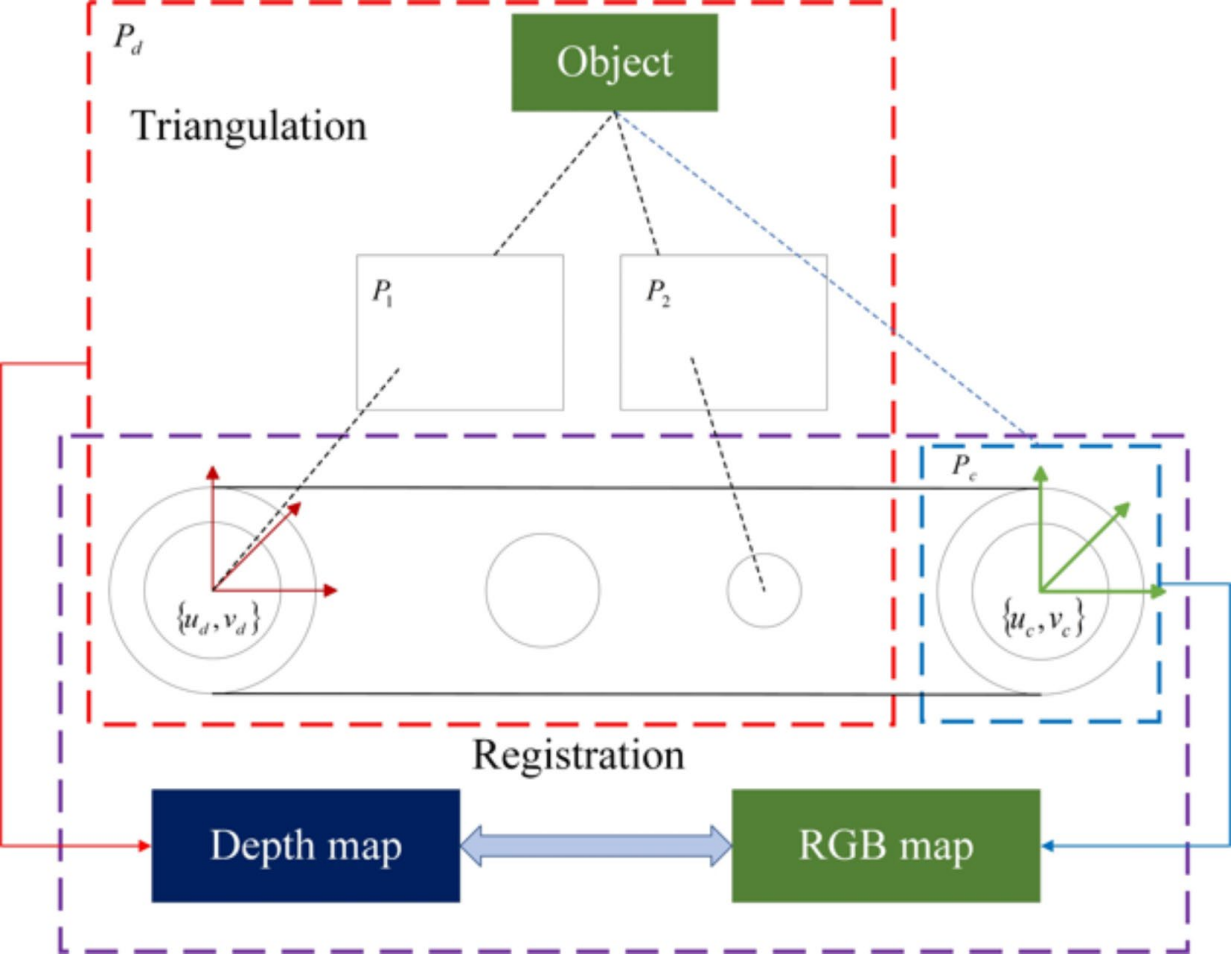
Camera registration diagram. (${P}_{1},{P}_{2}$ denote the infrared image taken by the left and right infrared cameras; ${P}_{d},{P}_{c}$ denote the depth map and color map respectively; $\left\{{u}_{c},{v}_{c}\right\}$ denotes the color pixel coordinate system; $\left\{{u}_{d},{v}_{d}\right\}$ denotes the depth pixel coordinate system).

As shown in Fig. 7, two infrared cameras are used to acquire depth images of the object from different angles, ${P}_{1}$ and ${P}_{2}$. The depth image ${P}_{d}$ can be aligned with the color image ${P}_{c}$ using the principle of triangulation. The pixel coordinate system $\left\{{u}_{c},{v}_{c}\right\}$ of the color image and the pixel coordinate system $\left\{{u}_{d},{v}_{d}\right\}$ of the depth image can then be determined. Once the alignment is complete, each pixel point $\left({u}_{c},{v}_{c}\right)$ in the color image corresponds to the pixel points $\left({u}_{d},{v}_{d}\right)$ in the depth image.

The depth camera simultaneously obtains the RGB image and depth information (point cloud map) of the target. The improved YOLOv5 analyzes the RGB image and outputs the predicted box of the target object. The center point of the diagonal of the predicted box is calculated as the object’s positioning point, and the point in the 3D point cloud corresponding to the positioning point is the depth information. So the distance between the object and the camera can be calculated.

### Validation method

Model validation is a crucial step in evaluating the performance of deep learning models, with the aim of assessing the model’s accuracy and its ability to generalize to unseen data. The primary goal of model validation is to observe the training and validation errors through the validation set, prevent overfitting, and inform further optimization of the model.

The accuracy of deep learning models on the training set is crucial and serves as a key indicator for measuring their generalization ability. For the YOLO model, other important metrics include recall rate, mean average precision (mAP), model size, and the number of parameters. The relevant calculation formulas are provided in Sect. 2.2. Ensure that the training and validation datasets are appropriately partitioned, and use the validation set to evaluate model performance in order to achieve optimal results. Some common strategies in deep learning for data partitioning include hold-out validation, K-fold cross-validation, and leave-one-out validation. The YOLO model uses the hold-out validation strategy to select its validation set. The validation set used in this study consisted of a self-collected dataset containing 1,852 indoor images, with an 8:2 hold-out validation ratio applied to ensure the random selection of images. The dataset includes indoor scenes with varying distances, low-light conditions, and instances of occlusion. In practical applications, the detection performance of YOLO models needs to be validated under diverse environmental conditions, including low-light settings, occlusion, and varying detection distances.

## Conclusions

To run the YOLO model effectively on portable mobile devices and accurately detect objects in complex indoor environments, we proposed an optimized version of the YOLOv5 model and developed an indoor object-finding system for the visually impaired based on portable mobile devices using the GhostNet network, CA mechanism, and BiFPN augmented features in conjunction with a RealSense D435i depth camera and Raspberry Pi device. The findings of this study can be summarized as follows:

(i) The YOLOv5s model was improved by replacing the backbone network with GhostNet to reduce the size of the model and the volume of data transmission. The CA mechanism was added, and the neck network was replaced with the BiFPN to increase the model precision. Compared to the original model, the model size was reduced by 42.4%, the number of parameters was reduced by 47.9%, and the recall was increased by 1.2%, with the same precision, enabling the model to perform better on portable mobile devices.
(ii) Accurate recognition and ranging of indoor objects was achieved using the RealSense D435i depth camera combined with a speech recognition and speech synthesis system to broadcast the exact locations of objects.
(iii) Using a Raspberry Pi 4 B device as the core processing unit, the improved YOLOv5 model, RealSense D435i depth camera, small amplifier module, and battery provided excellent portability and mobility.

## Data Availability

The complete Improved YOLOv5 model code is available via the following link. liushengCN/Improved-YOLO: Embeddable YOLO model (github.com).

## List of Symbols

${z}_{c}^{h}\left(h\right)$: Height ($h$); number of channels ($c$)
${z}_{c}^{w}\left(w\right)$: Width ($w$); number of channels ($c$)
$f$: Map of intermediate features of spatial information in the horizontal and vertical directions
$\delta\:$: Non-linear activation function
$${F}_{1}$$: Shared $$1\times\:1$$ convolution
$\sigma\:$: Sigmoid activation function
$${I}_{i}$$: Input feature
$$o$$: Output feature
$${\omega\:}_{n}$$: Learnable weights ($$n=i,j,\text{1,2},3$$)
$\epsilon$: A small amount to ensure stable output ($\epsilon =0.001$)
${P}_{6}^{td}$: Top-down intermediate feature
${P}_{6}^{out}$: Output feature from bottom to top
${O}_{c}$: Camera coordinate system origin
${O}_{i}$: Origin of the image coordinate system
$$\beta\:$$: Focal length
$\{x,y,z\}$: Coordinate system of the world
$\{{x}_{c},{y}_{c},{z}_{c}\}$: Coordinate system of the camera
$\{u,v\}$: Coordinate system of pixels
